# Usefulness of ultrasonography for the evaluation of cervical lymphadenopathy

**DOI:** 10.1186/1477-7819-9-29

**Published:** 2011-02-28

**Authors:** Rahul Khanna, Avinash Dutt Sharma, Seema Khanna, Mohan Kumar, Ram C Shukla

**Affiliations:** 1 Department of Surgery, Institute of Medical Sciences, Banaras Hindu University, Varanasi - 221 005, India; 2 Department of Pathology, Institute of Medical Sciences, Banaras Hindu University, Varanasi - 221 005, India; 3 Department of Radiodiagnosis, Institute of Medical Sciences, Banaras Hindu University, Varanasi - 221 005, India

## Abstract

**Aim:**

To evaluate the role of ultrasonography for differentiating cervical lymphadenopathy due to tuberculosis, metastasis and lymphoma.

**Methods:**

Ultrasonography of the neck nodes was carried out prior to FNAC in 192 patients using a 10 mHz linear transducer. The sonographic findings were then correlated with the definitive tissue diagnosis obtained by FNAC or lymph node biopsy.

**Results:**

The most significant distinguishing feature was strong internal echoes seen in 84% of tubercular lymph nodes. This finding was found in only 11% of metastatic nodes and absent in lymphomatous nodes. The other findings such as L/S ratio, irregular margins, hypoechoic center, fusion tendency, peripheral halo and absent hilus were helpful in differentiating reactive from diseased nodes but showed considerable overlap in the 3 groups of tubercular, metastatic and lymphoma lymph nodes.

**Conclusion:**

Ultrasonography is noninvasive and can give useful clues in the diagnosis of cervical lymphadenopathy. It should be interpreted in conjunction with FNAC result. Ideally ultra-sonographic guided FNAC should be obtained from the sonographically most representative node. In FNAC indeterminate cases, sonographic features may obviate the need for an invasive lymph node biopsy.

## Introduction

Cervical lymph nodes are frequently involved in a number of disease conditions. The most commonly seen causes of cervical lymphadenopathy are tuberculosis, distant metastasis and lymphoma. Fine needle aspiration cytology is used for evaluating enlarged cervical lymph nodes and has a high degree of sensitivity and specificity. However in our experience in almost 20% of patients FNACs may give an equivocal report, which would not contribute to the treatment. Ultrasonography has often been used to map out and characterize cervical lymph nodes specially for differentiating tubercular from malignant lymph nodes. The present study was designed to evaluate the role of ultrasonography for differentiating cervical lymphadenopathy of various causes.

## Materials and methods

The study was carried out over a 3 years period from January 2005 to December 2007 in the Department of Surgery, Banaras Hindu University. During this period, 204 patients with clinically palpable and untreated cervical lymph nodes visited our Out Patient Department. Out of these 192 patients were included in the study with tissue diagnosis of reactive lymph node, tubercular, metastatic or lymphoma involving cervical lymph nodes. Twelve patients were excluded as they had miscellaneous tissue diagnosis including bacterial, sarcoid and granulomatous lymphadenitis. There were 104 male and 88 female patients and the mean age was 47 years (range 8-71 years). These patients underwent an ultrasonographic examination of their neck nodes followed by FNAC of the most representative lymph node. The ultrasonographic findings were not available to the pathologist who performed the FNAC. The mean number of lymph nodes examined ultrasonographically per patient was 3.8 (range 1-9). In 34 patients (18%), the FNAC results were equivocal and a subsequent excisional biopsy of the lymph node was done to confirm the diagnosis. Correlation of the ultrasonographic findings with tissue diagnosis was done later.

### Method of ultrasonography

Ultrasonography was done using a 10 mHz linear transducer. The subject lay supine on the couch with the shoulders supported by a pillow and the neck hyper extended. Scans were obtained with the transducer placed transversely and longitudinally and measurements made in the plane that showed a maximum cross sectional area. Ultrasonographic characteristics were described as delineation of multiple lymph nodes, a tendency towards fusion, an internal echo, an irregular margin, the presence of strong echoes and posterior enhancement. Multiple lymph nodes were defined as involvement of more than 2 nodes. Fusion of lymph nodes was defined as partial or complete disappearance of a borderline echo between them. The internal echo of lymph nodes was assessed by the presence of hyperechoic echogenicity. Strong echoes were defined as single or multiple coarse high-echo spots located focally either in the central or peripheral area of the node. The shape of the lymph node was assessed by the L/S (long axis/short axis) ratio. An L/S ratio <2 indicates a round node whereas an L/S ratio >2 indicates an oval or elongated node.

### Method of FNAC

Fine Needle Aspiration Cytology (FNAC) of the most prominent node was carried out using a 22 Gauge needle attached to a 10 ml. syringe. Multiple passes using negative suction and through a single puncture site were done. This ensured that both the cortical and subcapsular areas of the node were sampled. Half of the aspirate was spread out onto a slide; air-dried and stained using MGG stain. The remaining half of the smear was wet fixed in alcohol-ether mixture and stained by Papanicolou stain. Two to three samples were obtained per lymph node and Cytotech was not used to check for adequacy of the sample.

The results of L/S ratio (long axis to short axis ratio) were expressed as a ratio of the respective sizes. The rest of the parameters were described as percentage positive out of the total number of lymph nodes examined. Statistical analysis and calculation of 'p values' was done by student's t test and Fisher's exact probability test.

## Results

Among the 192 patients a total of 730 lymph nodes were evaluated ultrasonographically. The various parameters studied were L/S ratio (long axis to short axis ratio) of lymph nodes, margins, hypoechoic center, fusion tendency, peripheral halo, absent hilus and strong internal echoes. Following ultrasonography, FNAC of the most prominent node was carried out. In 34 patients (18%), the FNAC result was equivocal and a subsequent excision biopsy of the lymph node was carried out to confirm the diagnosis. Core needle biopsy was not attempted as it is difficult to do on lymphnodes less than 1.5 cm in size and there is a risk of injury to underlying vascular structures. The final tissue diagnosis obtained on the basis of FNAC or biopsy was: Tubercular lymphadenopathy: 62(32%), metastatic deposit: 18(9%), lymphoma: 14(7%) and reactive lymph node: 98(51%). (Table 1).

**Table 1:** Ultrasonographic findings correlated with tissue diagnosis in cervical lymph nodes of 192 patients.

Characteristics	Tubercular (n = 62)	Metastatic (n = 18)	Lymphoma (n = 14)	Reactive (n = 98)	p value
L/S Ratio	1.8 ± 0.6	1.2 ± 0.3	1.5 ± 0.4	2.2 ± 0.9	<0.01

Irregular margins	41 (66%)	10 (55%)	3 (21%)	7(7%)	>0.01

Hypoechoic center	48 (77%)	11 (61%)	3 (21%)	8(8%)	>0.01

Fusion tendency	50 (81%)	12 (66%)	2 (14%)	Nil	>0.01

Peripheral halo	52 (84%)	10 (55%)	1 (7%)	Nil	<0.01

Internal echoe	52 (84%)	2 (11%)	Nil	Nil	<0.001

Absent hilus	16 (26%)	15 (83%)	4 (28%)	9(9%)	<0.01

The 'p values' compare the significance of difference between metastatic and lymphomatous nodes considered together versus the tubercular lymph nodes.

On ultrasonography, the long axis to short axis ratio of reactive lymph nodes was highest at 2.2 followed by 1.8 in tubercular, 1.5 in lymphoma and least in metastatic lymph nodes at 1.2 (p < 0.01). Fusion tendency, peripheral halo and internal echoes were not found in any of the reactive lymph nodes. Fusion tendency was found in 81% of tubercular, 66% of metastatic and 14% of lymphomatous nodes (p > 0.01). Significant differentiating feature among the 3 types of nodes were the presence of a peripheral halo and internal echoes. Peripheral halo was found in 84% of tubercular, 55% of metastatic and 7% of lymphoma nodes (p < 0.01). Internal echoes were reported in 84% of tubercular, 11% of metastatic and none of lymphoma nodes (p < 0.001).

Other ultrasonographic features such as an irregular margin, hypoechoic center and absent hilus were significant only as far as differentiating pathological from reactive lymph nodes were concerned. Among reactive nodes, irregular margins were found in 7%, hypoechoic center in 8% and absent hilus in 9% of nodes. The presence of irregular margins among tubercular nodes was 66%, metastatic nodes 55% and lymphoma nodes 21% (p > 0.01). Hypoechoic center was reported in 77% of tubercular, 61% of metastatic and 21% of lymphoma nodes (p > 0.01). Nodal hilus was absent in 26% of tubercular, 83% of metastatic and 28% of lymphoma nodes (p > 0.01).

## Discussion

Differentiation between tubercular, metastatic and lymphomatous cervical lymph nodes is extremely important from the therapeutic viewpoint. It is also important to make the correct diagnosis at the earliest because a delayed diagnosis can lead to upstaging of the malignancy making a curable lesion incurable. Clinicians have traditionally relied on FNAC to achieve a tissue diagnosis in cervical lymphadenopathy. The reported sensitivity and specificity of FNAC in the evaluation of cervical lymph nodes are 82% and 97% respectively [1]. However the FNAC report is frequently equivocal. Tubercular lymph nodes may be labeled as reactive or granulomatous lymphadenitis, which puts the treating doctor in a dilemma regarding starting anti tubercular treatment. Similarly in metastatic lymph nodes, sampling errors might occur because the lymph node chosen for FNAC may be reactive while the secondary deposit is harbored by other lymph nodes. Also FNAC is unreliable in differentiating between a metastatic and lymphomatous lymph node. Core needle biopsy is difficult to obtain from cervical lymph nodes. This is because of their small size, typically less than 1.5 cm. Trying to obtain a core needle biopsy especially with a tru cut needle from such small nodes puts the underlying vascular structures at risk of injury. The present study demonstrates the usefulness of ultrasonography used as an adjunct to FNAC in diagnosis of cervical lymphadenopathy.

The important ultrasonographic features of lymph nodes diagnosed on FNAC or histology to be reactive were a high L/S ratio (2.2) and absence of fusion tendency, peripheral halo and internal echoes. Irregular margins were found in only 7%, hypoechoic center in 8% and absence of hilus in 9% of reactive lymph nodes.

The ultrasonographic characteristics of tubercular lymphnode are said to be multiple lymph nodes, fusion tendency of adjacent nodes and a hypoechoic center with posterior enhancement. An additional feature, which has great specificity for tubercular lymphadenitis is strong echoes within the mass. The strong echoes are calcification within the node [2,3]. We found strong internal echoes in 84% (52/62) of tubercular lymphnodes and 11% (2/18) of metastatic lymph nodes. Internal echoes were absent in lymphomatous nodes.

Metastatic nodes are ultrasonographically characterized by a smaller long axis to short axis ratio (L/S ratio), absence of hilus and a hypoechoic center. We found that the mean L/S ratio of metastatic nodes was 1.2 ± 0.3, of lymphomatous nodes 1.5 ± 0.4 and tubercular lymph nodes 1.8 ± 0.6. An absent hilus was found in 83% (15/18) of metastatic nodes while only 26% (16/62) of tubercular and 28% (4/14) of lymphomatous nodes had absent hilus. This was because metastatic nodes tend to assume a more spherical shape. Steinkamp HJ et al report that 95% of metastatic nodes had L/S ratio of less than 2[4]. We also found a hypoechoic center in 61% (11/18) of metastatic lymph nodes which could reflect central necrosis. Fusion tendency was found in 66% (12/18) of metastatic nodes which could denote extra nodal spread and should be considered as a prognostic sign and also the need for post surgery adjuvant radiotherapy. Kim HC et al report the usefulness of 3 D ultrasonography for measuring volume of cervical lymph nodes. They found that a cut off volume of 0.7 cm^3 ^had a 80% sensitivity and 90% specificity for differentiating metastatic from reactive lymphadenopathy [5].

Doppler ultrasonography can evaluate the vascular pattern, displacement of vascularity, vascular resistance and pulsatility index. These features have been reported to have a sensitivity of 88% for the diagnosis of metastatic nodes and 67% for lymphoma with a specificity of 100%[6]. Metastatic lymph nodes are reported to have higher resistivity index (>0.8) and pulsatility index (>1.5) than reactive lymph nodes [7]. The limiting feature of Doppler and power ultrasound studies is their inability to distinguish between inflammatory and neoplastic nodes reliably on the basis of their flow pattern. Both metastatic and inflammatory nodes have associated vascularisation, which could appear similar on Doppler scan.

The main distinguishing feature of lymph nodes in lymphoma was a homogeneous pattern. In our study on 14 patients with lymphoma the mean L/S ratio was 1.5 ± 0.4, regular margin was seen in 79% (11/14) and none of them showed internal echoes within the lymph node. This could be attributed to the fleshy nature of these nodes and absence of either calcification or necrosis within them [8].

Ultrasonography is increasingly being recognized as a noninvasive tool for the evaluation of cervical lymph nodes. The sonographic appearance of normal nodes differs from those of abnormal nodes. Sonographic features, which help to identify abnormal nodes, are shape, absent hilus, intranodal necrosis, calcification, matting, peripheral halo and a prominent vascularity. A normal node should be discoid, with a hilus, sharp margins, absence of matting, calcification, necrosis or soft tissue edema. The sonographic features of matting are a tendency towards fusion, of calcification is a strong internal echo, of necrosis is an hypoechoic center and of soft tissue edema is a peripheral halo. Distinction between normal and abnormal cervical lymph nodes is fairly straightforward. However distinguishing tubercular from metastatic or lymphomatous lymph nodes is not very precise because of over lapping of many characteristics. The most significant distinguishing feature in our study was strong echoes within the node, which reflects intranodal calcification, caseation and granuloma formation. This was seen in 84% of tubercular, 11% of metastatic and none of the lymphomatous or reactive lymph nodes.

A low L/S ratio is found in lymph nodes, which have assumed a spherical shape. The lowest ratio of 1.2 ± 0.3 was seen in metastatic nodes, 1.5 ± 0.4 in lymphomatous, 1.8 ± 0.6 in tubercular and in reactive lymph nodes it was 2.2 ± 0.9. It is difficult to assign a cut off value separating the 3 categories because of overlap of cases, but this feature can be taken into consideration with other findings while arriving at a diagnosis. Other characteristics like irregular margin, hypoechoic center, fusion tendency and peripheral halo can be used to distinguish normal from abnormal nodes but are not of value in distinguishing between the 3 important causes of cervical lymphadenopathy.

## Conclusion

We conclude that ultrasonographic examination of cervical lymph nodes can yield important information regarding the diagnosis. The sonographic features should be used in conjunction with FNAC findings and may be especially helpful in cytologically indeterminate cases. Ultrasound examination should be done prior to FNAC and ideally an ultrasound guided FNAC sample should be obtained from the most sonographically representative node to reduce the sampling error. A lymph node biopsy can often be avoided by utilizing a combination of FNAC and ultrasonographic examination of the neck nodes.

## Competing interests

The authors declare that they have no competing interests.

## Authors' contributions

RK wrote the manuscript and supervised the work. ADS did the data collection and reviewed literature. SK did data analysis and writing of manuscript. MK did the FNAC. RCS did the ultrasonography. All authors read and approved the final manuscript.
